# RNA m^6^A methylation regulates virus–host interaction and EBNA2 expression during Epstein–Barr virus infection

**DOI:** 10.1002/iid3.396

**Published:** 2021-01-12

**Authors:** Xiang Zheng, Jia Wang, Xiaoyue Zhang, Yuxin Fu, Qiu Peng, Jianhong Lu, Lingyu Wei, Zhengshuo Li, Can Liu, Yangge Wu, Qun Yan, Jian Ma

**Affiliations:** ^1^ Hunan Cancer Hospital, The Affiliated Cancer Hospital of Xiangya School of Medicine Central South University Changsha Hunan China; ^2^ Cancer Research Institute, Department of Microbiology, Department of Pathology, School of Basic Medical Science Central South University Changsha Hunan China; ^3^ Department of Pathology Affiliated Hospital of Guilin Medical University Guilin Guangxi China; ^4^ Hunan Key Laboratory of Nonresolving Inflammation and Cancer, NHC Key Laboratory of Carcinogenesis Key Laboratory of Carcinogenesis and Cancer Invasion of Ministry of Education Changsha Hunan China; ^5^ Department of Immunology Changzhi Medical College Changzhi Shanxi China; ^6^ Department of Clinical Laboratory, Xiangya Hospital Central South University Changsha Hunan China

**Keywords:** BHRF1, EBNA2, Epstein–Barr virus, METTL3, RNA m^6^A methylation

## Abstract

**Introduction:**

N^6^‐methyladenosine (m^6^A) is the most prevalent modification that occurs in messenger RNA (mRNA), affecting mRNA splicing, translation, and stability. This modification is reversible, and its related biological functions are mediated by “writers,” “erasers,” and “readers.” The field of viral epitranscriptomics and the role of m^6^A modification in virus–host interaction have attracted much attention recently. When Epstein–Barr virus (EBV) infects a human B lymphocyte, it goes through three phases: the pre‐latent phase, latent phase, and lytic phase. Little is known about the viral and cellular m^6^A epitranscriptomes in EBV infection, especially in the pre‐latent phase during de novo infection.

**Methods:**

Methylated RNA immunoprecipitation sequencing (MeRIP‐seq) and MeRIP‐RT‐qPCR were used to determine the m^6^A‐modified transcripts during de novo EBV infection. RIP assay was used to confirm the binding of EBNA2 and m^6^A readers. Quantitative reverse‐transcription polymerase chain reaction (RT‐qPCR) and Western blot analysis were performed to test the effect of m^6^A on the host and viral gene expression.

**Results:**

Here, we provided mechanistic insights by examining the viral and cellular m^6^A epitranscriptomes during de novo EBV infection, which is in the pre‐latent phase. EBV *EBNA2* and *BHRF1* were highly m^6^A‐modified upon EBV infection. Knockdown of METTL3 (a “writer”) decreased EBNA2 expression levels. The emergent m^6^A modifications induced by EBV infection preferentially distributed in 3ʹ untranslated regions of cellular transcripts, while the lost m^6^A modifications induced by EBV infection preferentially distributed in coding sequence regions of mRNAs. EBV infection could influence the host cellular m^6^A epitranscriptome.

**Conclusions:**

These results reveal the critical role of m^6^A modification in the process of de novo EBV infection.

## Graphical abstract

The viral and cellular N^6^‐methyladenosine (m^6^A) epitranscriptomes were changed during de novo Epstein–Barr (EBV) infection in B cells. EBV EBNA2 was a significant m^6^A‐modified viral transcript, and cellular TLR9 and FAS genes' m^6^A modification levels are also modulated by EBV infection.

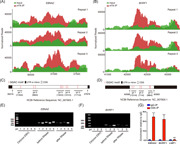

## 1 INTRODUCTION

N^6^‐methyladenosine (m^6^A) is the most prevalent modification that occurs in mammal messenger RNAs (mRNAs). Its related biological functions are mediated by “writers,” “erasers,” and “readers.” METTL3 and METTL14 serve as the most important catalytic subunits of the RNA methyltransferase complex, which catalytic writing of methyl groups into adenosines.^1^ The function of m^6^A is mediated partly by “reader” proteins, mainly identified in members of the YTH domain‐containing protein families.^2^ Besides mammals, m^6^A modification also occurs in the viral transcript, which is catalyzed by the methyltransferase system of the host cell, and thus to some extent affects the viral life cycle.^3^ m^6^A modification promoted replication in Simian virus 40^4^ and influenza A virus^5^ but attenuated the replication in hepatitis C virus^6^ and Zika virus.^7^ The effect of m^6^A modification on Kaposi's sarcoma‐associated herpesvirus (KSHV) replication was cell lines dependent.^8^ Tan et al.^9^ found that the cellular m^6^A/m epitranscriptome was reprogrammed during KSHV latent/lytic infection.

When Epstein–Barr virus (EBV) infects a human B lymphocyte, it goes through three phases: the pre‐latent phase, latent phase, and lytic phase (with appropriate stimuli).^10^, ^11^ Upon infection, EBV attaches to the receptors on the host cell surface, internalizes, and delivers the viral genome to the cell nucleus, followed by the circularization of the viral DNA and gradual acquisition of an epigenetic signature, including viral DNA nucleosome positioning and repressive chromatin mark introducing. The pre‐latent phase lasts for about 10 days, and later comes the latent phase. Upon appropriate stimuli such as 12‐*O*‐tetradecanoylphorbol‐13‐acetate and butyric acid, the lytic phase is induced, leading to virus synthesis.

In the current study, we were particularly concerned about whether m^6^A modification occurs in viral and cellular transcripts in de novo EBV‐infected cells, which is still in the pre‐latency phase. We found that EBV infection could influence the m^6^A methylation pattern of cellular transcripts. *EBNA2* and *BHRF1* contain m^6^A modifications in de novo EBV infection. We also found that the m^6^A machinery could modulate EBNA2 expression.

## 2 METHODS AND MATERIALS

### 2.1 Cell culture

BJAB (EBV‐negative B lymphoma cell line) and Raji (EBV‐positive B lymphoma cell line) cells were maintained in RPMI‐1640 (Hyclone) supplemented with 10% fetal bovine serum (FBS). Peripheral blood mononuclear cells (PBMCs) were isolated from whole blood of two health donors by Ficoll centrifugation. Primary B cells were isolated from PBMCs with CD19 microbeads (Miltenyl Biotec). All cell lines were obtained from the ATCC. The cell lines tested negative for mycoplasma contamination. All cell lines were authenticated by short tandem repeat profiling before use. Collections and use of blood samples were approved by the ethical review committees of the appropriate institutions.

### 2.2 EBV virus preparation and infection

Infectious EBV was produced from the B95.8 cell culture supernatants. Briefly, B95.8 cells were prepared in RPMI‐1640 medium containing 10% FBS. Cells were centrifuged and started at a new culture at 2 × 10^5^/ml density in RPMI‐1640 medium containing 2% FBS. Cells were cultured at 37°C with 5% CO_2_ for 2 weeks without changing the medium. Cells were centrifuged at 300*g* to sediment cells and debris, passed through 0.45‐μm Millipore filters, then further centrifuged at 50,000*g* at 4°C, and resuspended in fresh FBS‐free RPMI‐1640. To determine the multiplicity of infection (MOI) of EBV, a DNA Quantitative Fluorescence Diagnostic Kit (Sansure Biotech) was used according to the manufacturer's protocol and the published literature.^12^ In this study, we used 50 MOI EBVs to infect BJAB cells unless otherwise indicated.

### 2.3 Lentivirus transduction

Short hairpin (shRNA) lentiviruses were obtained from GenePharma. Lentiviral vector plasmids LV3 (H1/GFP&Puro) were used in this study to construct stable cell lines. Lentiviruses were transduced into cells according to the manufacturer's instructions. The shRNA sequences are listed in Table S1.

### 2.4 Western blot analysis

Protein extracts were resolved by sodium dodecyl sulfate–polyacrylamide gel, transferred to polyvinylidene fluoride membranes, and probed with antibodies against METTL3 (Cat# 15073‐1‐AP; Proteintech), TLR9 (Cat# 13674; Cell Signaling Technology), FAS (#4233; Cell Signaling Technology), EBNA2 (Cat # MABE8; Millipore), GAPDH (Cat# D110016; Sangon Biotech). Horseradish peroxidase (HRP)‐conjugated AffiniPure goat anti‐rat IgG (Cat# SA00001‐15; Proteintech), anti‐rabbit IgG HRP‐linked antibody (Cat# 7074; Cell Signaling Technology) was used as the secondary antibody. Glyceraldehyde‐3‐phosphate dehydrogenase (GAPDH) was used as an internal loading control.

### 2.5 Quantitative reverse‐transcription polymerase chain reaction (RT‐qPCR) analysis

Total RNAs were extracted using Trizol (Invitrogen). For mRNA reverse transcription, 2 μg of RNA was used to synthesize complementary DNA (cDNA) using a Maxima H Minus First Strand cDNA synthesis kit with dsDNase (Thermo Fisher Scientific) according to the manufacturer's protocol. The levels of gene transcripts were detected by quantitative polymerase chain reaction (qPCR) using specific primers and an SYBR premix Ex TaqII Kit (Takara). The expression levels of mRNA were quantified by measuring cycle threshold (*C*_t_) values and normalized to *ACTIN*. The data were further normalized to the negative control unless otherwise indicated. The primers used for RT‐qPCR are listed in Table S2.

### 2.6 Methylated RNA immunoprecipitation sequencing (MeRIP‐seq) and data analysis

BJAB cells were infected with 50 MOI EBVs for 24 h. The uninfected cells were used as a negative control. Total RNAs were extracted from BJAB cells. Intact mRNA was isolated from total RNAs using the Arraystar Seq‐Star™ poly(A) mRNA Isolation Kit according to the manufacturer's protocol, then the isolated mRNA was chemically fragmented to 100‐nucleoside‐long fragments by incubation in the fragmentation buffer (10 mM Zn^2+^ and 10 mM Tris‐HCl, pH 7.0). The m^6^A methylated mRNAs were immunoprecipitated with anti‐m^6^A antibody (#202003; Synaptic Systems) and one‐tenth of the fragmented mRNAs was kept as input. The major procedures contained immunoprecipitation, washing, and elution. The eluted mRNA fragments were concentrated for RNA‐seq library construction. RNA‐seq libraries for the m^6^A antibody‐enriched mRNAs and input mRNAs were prepared using the KAPA Stranded mRNA‐seq Kit (Illumina). The prepared libraries were diluted to 8 pM and clusters were generated on the Illumina cBot using a HiSeq 3000/4000 PE Cluster Kit. Sequencing was performed using the Illumina HiSeq 4000. Raw data were trimmed using Trimmomatic (v0.32) and aligned to Ensembl reference genome and EBV reference genome (NC_007605.1) using HISAT2 software (v2.1.0). Peak calling and differentially methylated peaks analyzing were performed using the exomePeak (v2.13.2) as described.^13^ For differential methylated peaks analysis, a fold change of minimally 1.5 and a maximum *p* value of .05 were considered significantly differential between the two groups to explore as many differentially methylated peaks as possible. The peaks were annotated according to the annotation information in Ensembl database. The EBV reference genome and annotation were downloaded from https://www.ncbi.nlm.nih.gov/nuccore/NC_007605.1. The peaks were visualized in Integrated Genome Viewer (IGV). The raw sequencing data obtained from the MeRIP‐seq reported in this study have been deposited in NCBI GEO under accession No. GSE133936.

### 2.7 MeRIP‐RT‐qPCR

This procedure was adapted from the published reports.^14^, ^15^, ^16^ Briefly, intact poly (A) + RNAs from cells were isolated by using a Magnetic mRNA Isolation Kit (New England Biolabs) according to the manufacturer's protocol, but not randomly fragmented to facilitate reverse transcription with oligo(dT) and PCR amplification. mRNAs were incubated with 5 μg of anti‐m^6^A antibody (#202003; Synaptic Systems) for 2 h at 4°C in IP buffer (150 mM NaCl, 10 mM Tris‐HCl, 0.1% NP‐40, pH 7.4) containing RNase inhibitor (Promega). IP with normal rabbit IgG were performed in parallel. The mixture was then incubated with protein A/G magnetic beads (Selleck) at 4°C for 2 h. After washing for three times with IP buffer, the bound RNAs were eluted from the beads in IP buffer containing 6.7 mM m^6^A sodium salt (Santa Cruz Biotechnology) and ethanol precipitated. cDNA synthesis and qPCR analysis were performed as described above.

### 2.8 RNA immunoprecipitation (RIP) assays

Briefly, Raji cells were transfected with Flag‐YTHDF1 or ‐YTHDF2 or ‐YTHDF3 plasmids for 48 h. Then, the cells were harvested and washed twice in ice‐cold phosphate‐buffered saline (PBS). The cell pellet was resuspended in gentle lysis buffer containing RNase inhibitor (Promega) and protease inhibitor (Selleck), and incubated on ice for 20 min. After centrifugation at 12,000 rpm at 4°C for 15 min, the supernatants were incubated with anti‐Flag antibody (#F1804; Sigma‐Aldrich) or control anti‐IgG antibody (#sc‐2025; Santa Cruz Biotechnology) conjugated to protein A/G magnetic beads (Selleck) and rotated at 4°C for 4 h. The supernatant was removed, and the beads were washed extensively by wash buffer, followed by adding 0.5 ml of Trizol. cDNA synthesis and qPCR analysis were performed as described above.

### 2.9 RNase‐mediated RNA–protein interaction determining assay

This procedure was adapted from the published reports.^17^, ^18^, ^19^ Briefly, BJAB cells were infected with EBV for 24 h. One‐tenth of the sample was saved for RNA purification and used as the “before RNase treatment.” Cells were treated with 1% formaldehyde at room temperature with shaking for 10 min. A final concentration of 125 mM glycine was then added dropwise for additional 5‐min incubation. Then, cells were washed twice with ice‐cold PBS and collected by centrifugation. The pellets were resuspended by sonication in 900 μl gentle lysis buffer containing DNase I (2 U/ml) and a protease inhibitor cocktail, followed by 100 ng/ml RNase incubation at 37°C for 30 min. In total, 100 μl of the sample was saved as the “after RNase treatment.” Then RNAs were extracted. cDNA synthesis was performed using reverse transcriptase and random primers. qPCR analysis was done as described above. The primers are listed in Table S2.

### 2.10 RNA decay assay

This procedure was adapted from the published reports.^20^ BJAB cells were plated on 12‐well plates with 5 × 10^5^ cells per well. Actinomycin‐D (Meilunbio) was added to a final concentration of 5 μg/ml, and cells were collected before or 4 h after adding Actinomycin‐D. Then, the cells were processed as described in “RT‐qPCR,” except that the data were normalized to before Actinomycin‐D treatment.

### 2.11 Statistical analysis

Statistical significance was calculated using Prism (GraphPad Software) and SPSS17. All experiments were performed in triplicate. Data represent the mean ± *SD*. Statistical differences were assessed with the unpaired Student *t* test, and *p*s < .05 were considered to reflect statistical significance. A one‐way analysis of variance test was performed for comparing three or more groups within the same experiment. In all results, NS denotes “not significant,” *p < .05, **p < .01, and ***p < .001 compared with the indicated control group.

## 3 RESULTS

### 3.1 EBV infection influences m^6^A methylation pattern of cellular transcripts

**Figure 1:**
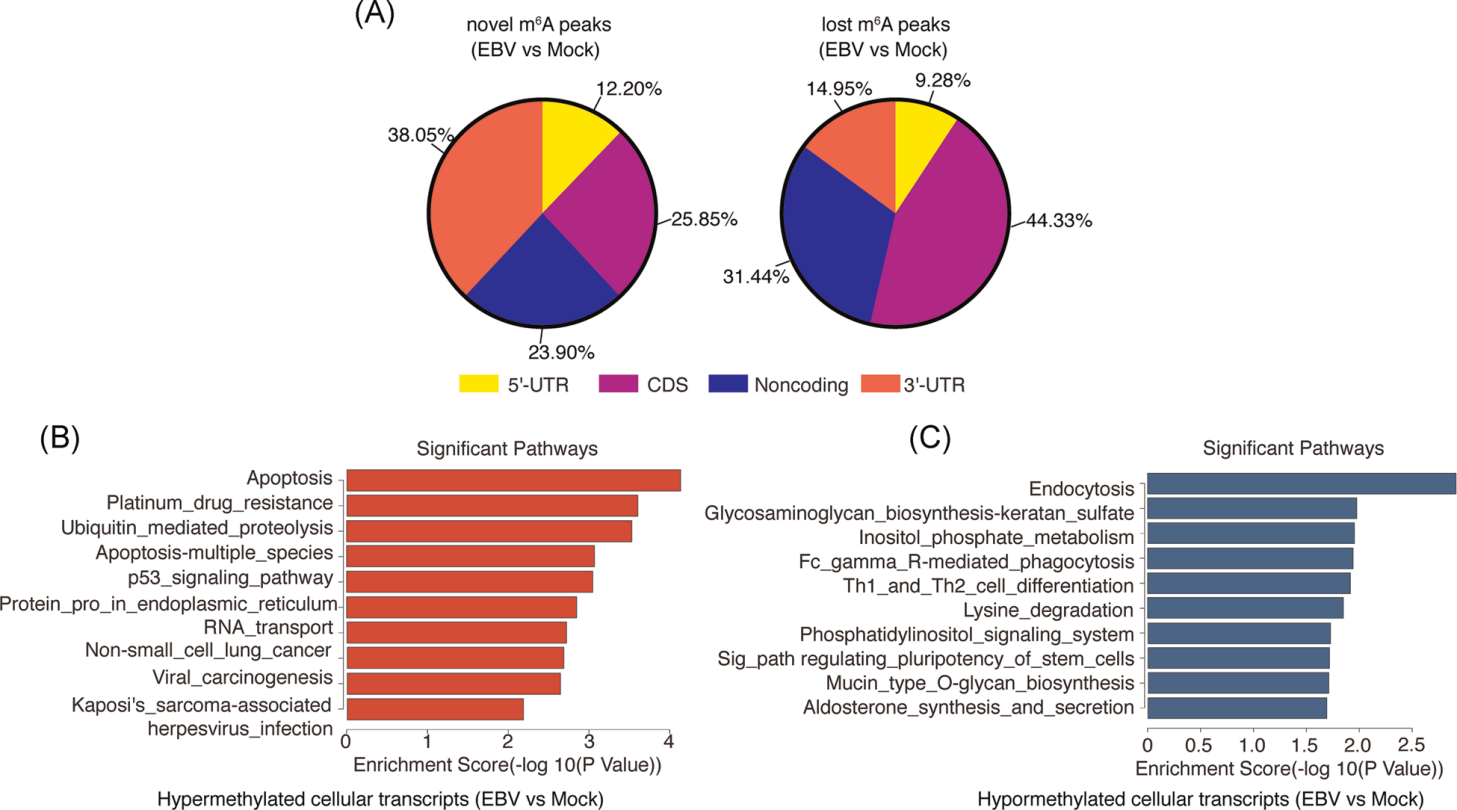
EBV infection influences m^6^A methylation pattern of cellular transcripts. MeRIP‐seq of BJAB cells which were infected by EBV (or uninfected as a negative control, i.e., “mock”) for 24 h. (A) Distribution pattern of newly emergent m^6^A peaks (left) or loss of existing m^6^A peaks (right) upon EBV infection. (B) KEGG analysis of pathways enriched in the hypermethylated genes induced by EBV infection (i.e., EBV infection vs. mock). The top 10 enriched pathways are shown. (C) KEGG analysis of pathways enriched in the hypomethylated genes induced by EBV infection. Top 10 enriched pathways are shown. CDS, coding sequence; EBV, Epstein–Barr virus; KEGG, Kyoto Encyclopedia of Genes and Genomes; UTR, untranslated region

To determine whether EBV infection could modulate the cellular epitranscriptome, we examined the human BJAB cell line at 24 h post‐EBV infection by methylated RIP sequencing (MeRIP‐seq) experiments. To confirm the successful establishment of EBV infection, we determined the mRNA expression levels of *EBNA2* and *EBNA‐LP*, two of the first expressed viral genes during de novo EBV infection.^21^ *EBNA2* and *EBNA‐LP* were detected in EBV‐infected samples but not in uninfected samples (shown in Figure S1), which confirmed the success of the virus infection. We analyzed the distribution pattern of cellular m^6^A peaks and found that the newly “gained” (i.e., novel) m^6^A peaks were preferentially deposited in 3ʹ untranslated region (UTR) of the cellular transcripts, whereas the “lost” m^6^A peaks were preferentially distributed in coding sequence (CDS) regions of the cellular transcripts (shown in Figure 1A). There were 918 significantly upregulated m^6^A peaks (from 416 genes), and 2586 significantly downregulated m^6^A peaks (from 1046 genes) induced by EBV infection (i.e., EBV infection vs. mock; Figure S2; NCBI GEO data #GSE133936). We then performed a Kyoto Encyclopedia of Genes and Genomes (KEGG) analysis of these genes. The apoptosis pathway and endocytosis pathway ranked first in the enriched pathways of hypermethylated genes and hypomethylated genes, respectively (EBV infection vs. mock, shown in Figure 1B,C). These findings suggest that de novo EBV infection could to some extent alter the host cellular N^6^‐methyladenosine epitranscriptome.

### 3.2 EBV infection modulates FAS and TLR9 m^6^A methylation levels and expression

**Figure 2:**
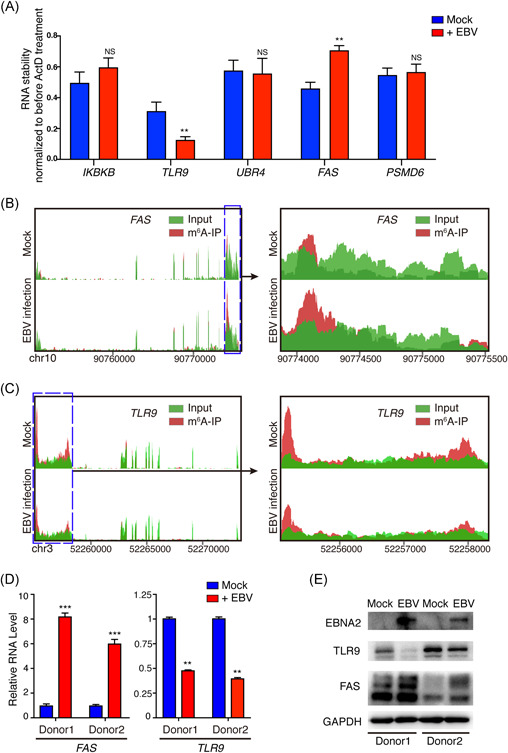
EBV infection modulates the cellular transcripts m^6^A methylation levels. (A) BJAB cells were infected with EBV (or mock infection as negative control) for 24 h, then the cells were treated with Act‐D for 4 h. The RNAs were extracted before and 4 h after adding Act‐D, and determined by RT‐qPCR. Each column represents the relative mRNA levels (i.e., after 4‐h Act‐D treatment vs. before Act‐D treatment). The *y*‐axis value represents the mRNA stability of an indicated gene under two situations (mock or EBV infection). (B, C) The representative pictures of visualization of methylated RNA immunoprecipitation sequencing results show *FAS* (B) and *TLR9* (C) regions of enrichment for m^6^A immunoprecipitation (red) over input (green) for BJAB cells (24 h post EBV or mock infection). (D) Primary B cells from two healthy donors were infected with 10 MOI EBV for 36 h. The *FAS* and *TLR9* mRNAs levels were analyzed by RT‐qPCR. (E) Primary B cells were infected with 10 MOI EBVs for 48 h. Cell lysate was analyzed by Western blot analysis. *ACTIN* was used as internal control for RT‐qPCR. GAPDH was used as internal control for Western blot analysis. Values are the mean ± *SD* (*n* = 3). NS, not significant; *p < .05, **p < .01, ***p < .001 comparing to control. Act‐D, actinomycin‐D; EBV, Epstein–Barr virus; GAPDH, glyceraldehyde‐3‐phosphate dehydrogenase; MOI, multiplicity of infection; mRNA, messenger RNA; RT‐qPCR, quantitative reverse‐transcription polymerase chain reaction

MeRIP‐seq showed that EBV infection altered the host N^6^‐methyladenosine epitranscriptome. To determine the effect of EBV infection on host cellular genes, we chose some hypermethylated or hypomethylated genes for further study, which mainly were involved in apoptosis and the immune system. Of these genes, m^6^A peaks of *UBR4, FAS*, and *PSMD6* exhibited increased abundance upon EBV infection, whereas *IKBKB* and *TLR9* were decreased (shown in Table S3). As m^6^A modification plays an important role in modulating mRNA stability, we tried to determine the role of EBV infection in regulating these mRNAs' stability. EBV infection enhanced the mRNA stability of *FAS*, whereas it repressed the mRNA stability of *TLR9* (Figure 2A). *FAS* is related to apoptosis pathway,^22^ and *TLR9* is related to virus infection,^23^ both of them are involved in pathogenesis of EBV,^24^, ^25^ we chose *FAS* and *TLR9* for further study. MeRIP‐seq showed that m^6^A abundance was increased in *FAS* mRNA transcripts (shown in Figure 2B) whereas decreased in *TLR9* mRNA transcripts (shown in Figure 2C) upon EBV infection, suggesting that EBV could modulate their m^6^A modification levels. We also extracted primary B cells from two healthy donors, and the cells were infected with 10 MOI EBVs. We found that the mRNA and protein levels of FAS were upregulated, whereas TLR9 were downregulated after EBV infection (shown in Figure 2D,E).

### 3.3 EBV *EBNA2* and *BHRF1* are m^6^A‐modified during de novo EBV infection

**Figure 3:**
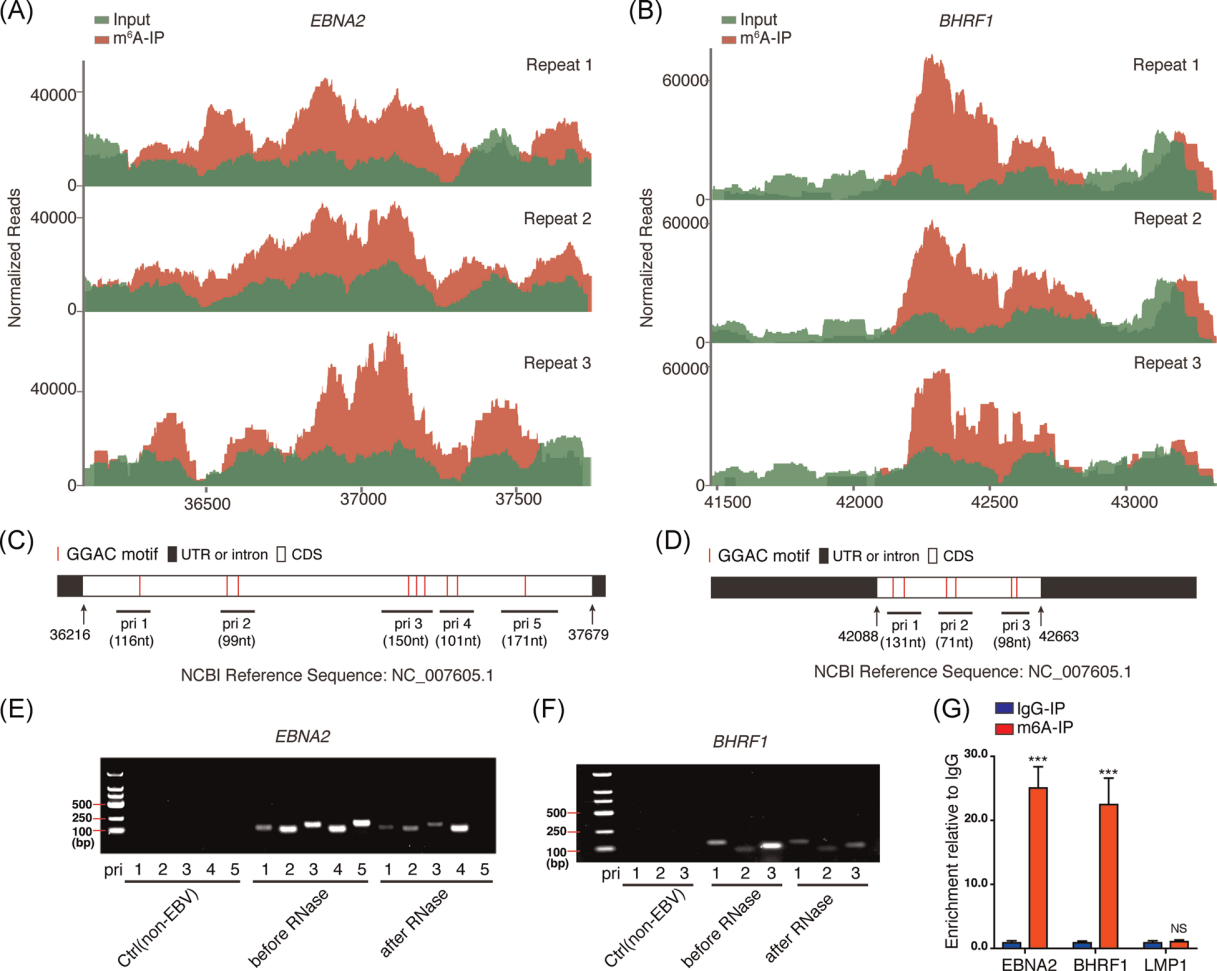
EBV *EBNA2* and *BHRF1* messenger RNAs contain m^6^A modifications. (A, B) Visualization of MeRIP‐seq shows *EBNA2* (A) and *BHRF1* (B) transcripts contain m^6^A modifications in EBV‐infected BJAB cells (24‐h post‐infection). Reads were normalized to the total number of reads mapping to the viral genome. (C, D) Schematic presentation of m^6^A “GGAC” motif sequences located in *EBNA2* (C) and *BHRF1* (D) CDS. The amplified fragments contained “GGAC” motifs by the indicated primers (pri) are shown. (E, F) RNA from BJAB cells (24‐h post‐infection) were extracted before or after formaldehyde cross‐linking followed by RNase treatment. The products amplified by the indicated primers were analyzed in an 2% agarose gel by electrophoresis. Cells without EBV infection were used as negative control. (G) MeRIP‐RT‐qPCR. RNAs were harvested from EBV‐infected BJAB cells and immunoprecipitated with anti‐m^6^A (or IgG as negative control). Eluted RNAs from the immunoprecipitation were quantified as a percentage of input. The enrichment values of “IgG‐IP” group are set at 1 for *EBNA2, BHRF1*, and *LMP1*. Values are the mean ± *SD* (*n* = 3). ***p < .001 comparing to “IgG‐IP” group. CDS, coding sequence; EBV, Epstein–Barr virus; IgG, immunoglobulin G; MeRIP‐seq, methylated RNA immunoprecipitation sequencing; RT‐qPCR, quantitative reverse‐transcription polymerase chain reaction; UTR, untranslated region

To determine whether viral transcripts are m^6^A‐modified during the course of de novo EBV infection, the MeRIP‐seq reads in the EBV‐infected BJAB cells were aligned to EBV reference genome (https://www.ncbi.nlm.nih.gov/nuccore/NC_007605.1). Two EBV transcripts, *EBNA2* and *BHRF1*, were found containing m^6^A modifications, which are located in their CDS regions. The results of the three repeats were highly consistent (shown in Figure 3A,B). The *EBNA2* CDS region contained nine “GGAC” motifs (shown in Figure 3C), while *BHRF1* CDS contained six “GGAC” motifs (shown in Figure 3D), the canonical m^6^A motif. According to the distribution of these “GGAC” motifs in *EBNA2* and *BHRF1* CDS, we designed several pairs of primers (shown in Figure 3C,D and Table S2), and performed formaldehyde cross‐linking followed by RNase‐mediated experiments to evaluate whether these mRNA regions are protected by binding proteins (e.g., m^6^A “readers”). The amplified regions from primers 1–4 but not 5 in *EBNA2* and 1–3 in *BHRF1* were protected in varying degrees against the degradation of RNase (shown in Figure 3E,F). Considering the m^6^A motifs' location, the results indicated these regions (i.e., primers 1–4 for *EBNA2*, and primers 1–3 for *BHRF1*) were probably bound by m^6^A binding proteins. To further validate m^6^A modification in the two viral mRNAs, we performed MeRIP‐RT‐qPCR to detect enriched RNAs after anti‐m^6^A immunoprecipitation. *EBNA2* and *BHRF1*, but not *LMP1* RNAs, were specifically enriched by the anti‐m^6^A antibody (immunoglobulin G [IgG], was served as a negative control for anti‐m^6^A antibody). *LMP1* is an EBV‐encoded oncogene. No m^6^A‐modified *LMP1* transcript was detected from BJAB cells at 24‐h post‐EBV infection (shown in Figure 3G). These results suggested that viral genes *EBNA2* and *BHRF1* are m^6^A‐modified during de novo EBV infection.

### 3.4 METTL3 and YTHDFs regulate the expression of EBNA2

**Figure 4:**
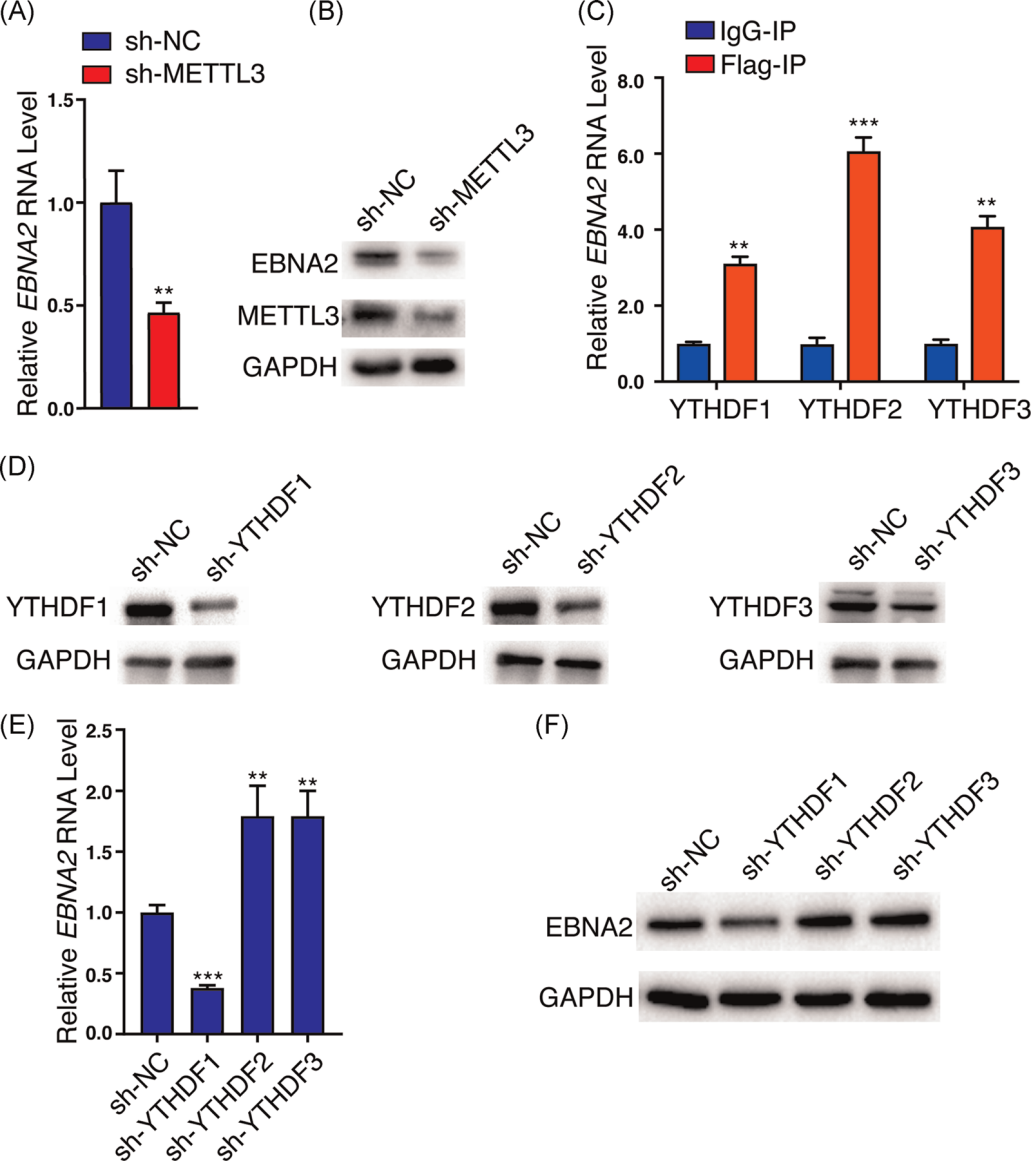
Expression of endogenous EBNA2 in Raji cells is regulated by cellular m^6^A machinery. (A, B) EBNA2 mRNA (A) and protein (B) levels of Raji cells (sh‐NC and sh‐METTL3) were assayed by RT‐qPCR and Western blot analysis, respectively. (C) The association between *EBNA2* mRNA and “readers.” RIP from Flag‐YTHDF1 or ‐YTHDF2 or ‐YTHDF3 expressed Raji cells using an anti‐Flag antibody or IgG (as negative control), with RT‐qPCR analysis of *EBNA2*, were quantified as the percent of input. The enrichment values of “IgG‐IP” group are set at 1. (D) The endogenous expression of YTHDF1, YTHDF2, or YTHDF3 in Raji cells were knocked down by lentivirus carrying short haipin RNAs targeting YTHDF1, YTHDF2, or YTHDF3, respectively. Western blot analysis of proteins harvested from Raji cells. GAPDH was used as internal control. (E, F) The EBNA2 mRNA (E) and protein (F) levels of Raji cells (sh‐NC, sh‐YTHDF1, ‐YTHDF2, or ‐YTHDF3) were assayed by RT‐qPCR and Western blot analysis, respectively. Values are the mean ± *SD* (*n* = 3). **p < .01, ***p < .001 comparing to control. Relative levels of EBNA2, METTL3, or YTHDF proteins in each group compared with the sh‐NC group are indicated. GAPDH, glyceraldehyde‐3‐phosphate dehydrogenase; IgG, immunoglobulin G; mRNA, messenger RNA; RIP, RNA immunoprecipitation; RT‐qPCR, quantitative reverse‐transcription polymerase chain reaction

Given the significant deposition of m^6^A peaks in *EBNA2* mRNA, we asked whether m^6^A modification can regulate *EBNA2* expression. The reversible addition and removal of m^6^A from mRNAs are thought to be dynamically regulated. The m^6^A “writer” METTL3 is a major component of the methyltransferase complex required for m^6^A modification, and we determined the effect of METTL3 on *EBNA2* expression. We constructed the Raji cell (an EBV‐positive B lymphoma cell line) with METTL3 knockdown by means of lentivirus carrying shRNA and found that knockdown of METTL3 inhibited endogenous EBNA2 mRNA and protein expression (shown in Figure 4A,B). Meanwhile, knockdown of FTO, an m^6^A “eraser,” significantly increased the EBNA2 protein levels (Figure S3). The fate of m^6^A‐modified mRNA is mainly mediated by m^6^A “readers,” which recognize the site of m^6^A modification. Several proteins with the YTH domain were shown to bind m^6^A‐modified RNAs. We thus evaluated the effects of “readers” YTHDF1, YTHDF2, and YTHDF3 on *EBNA2* expression. We determined the binding ability of the three YTHDFs to *EBNA2*. As the RIP grade antibodies for YTHDF1, YTHDF2, and YTHDF3 are not commercially available, pulling down the endogenous YTHDFs is not feasible. We transfected the Raji cells with Flag‐tagged YTHDF1, YTHDF2, or YTHDF3 plasmids for 48 h. RIP assay showed that *EBNA2* RNAs were specifically enriched by the anti‐Flag antibody compared with IgG, suggesting all the three readers can bind to *EBNA2* mRNA (shown in Figure 4C). We knocked down YTHDF1, YTHDF2, or YTHDF3 in Raji cells with lentivirus carrying shRNAs, respectively (shown in Figure 4D). The mRNA and protein levels of endogenous EBNA2 were decreased when YTHDF1 was knocked down but increased when YTHDF2 or YTHDF3 was knocked down in Raji cells (shown in Figure 4E,F). Meanwhile, exogenous expression of YTHDF1 increased the EBNA2 protein levels (Figure S4). These results implied that METTL3 and YTHDFs exert a different influence on EBNA2 expression, METTL3 and YTHDF1 increased EBNA2 expression, whereas YTHDF2 and YTHDF3 decreased its expression.

## 4 DISCUSSION

Recently, a study from Robertson's group that focused on the role of m^6^A modification in EBV latent and lytic phases was published.^26^ They found knockdown of *METTL14* led to decreased expression of latent EBV transcripts and demonstrated that EBNA3C activated *METTL14* transcription and increased its stability, contributing to EBV‐mediated tumorigenesis. Their findings provided important resources for understanding m^6^A modification in EBV latent and lytic phases. However, little is known about the role of m^6^A modification during EBV de novo infection. In the current study, we employed a system of infecting B cells with EBV and investigated the virus–host interaction in the de novo infection phase from the perspective of m^6^A modification.

In de novo EBV‐infected BJAB cells, the most apparent changes in host N^6^‐methyladenosine epitranscriptome were the emerged m^6^A modifications preferentially distributed in the 3ʹ‐UTR region of cellular transcripts, while the lost m^6^A modifications preferentially distributed in CDS (shown in Figure 1A). mRNA m^6^A modification located in the 3ʹ‐UTR and CDS regions may result in different outcomes recognized by different m^6^A “readers.” For example, METTL3 and YTHDF1 preferentially recognize m^6^A residues on CPCP1 3ʹ‐UTR and promote CDCP1 translation.^27^ Wu et al.^28^ found that JAK2 and SOSC3 have m^6^A modification at 3ʹ‐UTR and demonstrated that YTHDF1 could bind m^6^A‐modified mRNA of JAK2 to promote translation and protein expression, while YTHDF2 could target m^6^A‐modified mRNA of SOCS3 to reduce the protein abundance. Mao et al.^29^ demonstrated that m^6^A in mRNA CDS regions promoted translation and removing CDS m^6^A results in a further decrease of translation. Li et al.^30^ found that methylated SOX2 transcripts, specifically the CDS regions, could be recognized by IGF2BP2 to prevent SOX2 mRNA degradation. These results implied that EBV infection might modulate host mRNA stability, translation or protein expression through altering the distribution pattern of m^6^A modification. Among the enriched pathways of m^6^A hypomethylated cellular genes, the endocytosis pathway ranked first (shown in Figure 1C). As receptor‐mediated endocytosis is the main way for EBV entry into susceptible cells, this result may imply that m^6^A modification may be involved in the process of EBV entry into the host cells. Some molecule metabolic pathways were enriched in m^6^A hypomethylated cellular genes, including inositol phosphate metabolism and lysine degradation, suggesting m^6^A modification may be involved in the process of EBV infection‐associated metabolism dysfunction. In the m^6^A hypermethylated cellular genes upon EBV infection, some important signaling pathways including apoptosis, ubiquitin‐mediated proteolysis, and viral carcinogenesis were enriched (shown in Figure 1B). Considering the effect of m^6^A modification on gene expression, these results suggested that EBV infection may modulate some gene expression by altering their m^6^A modification. However, the specific mechanism needs to be further studied. One of these genes is *FAS*, an important gene of the apoptosis pathway.^22^ The stability and expression of *FAS* mRNA were enhanced by EBV infection (shown in Figure 2). It was reported that EBV LMP1 and LMP2A can induce *FAS* expression,^31^, ^32^ and de novo EBV infection also can increase *FAS* expression in T cells.^33^ Our observation suggested that besides LMP1 and LMP2A, EBV infection may upregulate the *FAS* expression at least partly by increasing its m^6^A modification levels (shown in Figure 2).  Toll‐like receptor (TLR) signaling is responsible for the primary recognition of infectious agents leading to the initiation of the innate and adaptive immune response. Among the TLRs, TLR9 senses unmethylated CpG double‐stranded DNA (dsDNA) motifs.^34^, ^35^ It is conceivable that EBV is sensed by TLR9 in B cells during their de novo infection, because of the unmethylated dsDNA. However, EBV can suppress TLR9 expression to evade innate immune recognition and benefit the long‐term survival of the virus.^23^, ^36^ Consistent with these studies, we found that EBV de novo infection suppressed the expression of *TLR9*. Given that the m^6^A modification and mRNA stability of *TLR9* were significantly reduced after EBV infection (shown in Figure 2), m^6^A modification might enhance *TLR9* mRNA stability. EBV may suppress TLR9 signaling by decreasing the m^6^A modification of the *TLR9* transcript, and this might be a strategy employed by the virus to evade immune surveillance. Manipulating the m^6^A modification level of *TLR9* may be a new therapeutic target.^37^, ^38^

In Robertson lab's study,^26^ they comprehensively defined much m^6^A modification of EBV latent and lytic transcripts. In the current study, we found that two EBV transcripts, *EBNA2* and *BHRF1*, were m^6^A‐modified when BJAB cells were infected with EBV for 24 h (shown in Figure 3A,B). These differences may be due to the different expression patterns of EBV genes in different life cycles. The background of our study is in the pre‐latent phase during EBV de novo infection. It is reasonable that *EBNA2* was m^6^A‐modified during EBV de novo infection because as early as 6‐h post‐infection, *EBNA2* transcripts were detectable in de novo‐infected B lymphocytes.^21^ The lytic early gene *BHRF1* was also m^6^A‐modified in de novo‐infected cells. The expression of *BHRF1* might result from the epigenetically “naked” EBV genome that was not introduced to the repressive chromatin marks in the de novo‐infected cells. In fact, Altmann et al.^39^ demonstrated that *BHRF1* expression reached a high level at 24‐h post‐EBV infection and then declined rapidly. Our study also suggested the expression of *BHRF1* at 24 h in EBV de novo infection. We chose *EBNA2* for further study because of its essential role in B‐cell proliferation, immortalization, activation of super‐enhancers,^40^ and recently we reported EBNA2 can form liquid‐like condensates through phase separation at super‐enhancer sites of MYC and Runx3.^41^ Our results suggested EBNA2 was precisely regulated by the host m^6^A machinery. Figure 3E suggested that *EBNA2* RNA was protected by m^6^A binding proteins; Figure 4A,B revealed that METTL3 increased the expression of EBNA2 in Raji cells. Previous reports suggested that YTHDF1, YTHDF2, and YTHDF3 could function cooperatively to promote efficient translation or degradation of specific m^6^A‐containing mRNAs.^42^, ^43^, ^44^ In a further study, we also found that EBNA2 expression was regulated by these m^6^A “readers.” YTHDF1, YTHDF2, and YTHDF3 could bind m^6^A‐modified *EBNA2* mRNA. YTHDF1 increased EBNA2 expression, whereas YTHDF2 and YTHDF3 decreased its expression (shown in Figure 4E,F), which implied the importance of the reader's selection and binding. EBNA2 is one of the earliest expressed genes in EBV‐infected B lymphocytes, the viral *Cp* and *LMP1, LMP2A*, and *LMP2B* promoters are strongly activated by EBNA2. Our study confirmed that the m^6^A machinery could regulate the expression of EBNA2, which may affect the function of EBNA2 as a transcriptional activator, and further affect the expression pattern of virus genes and the life cycle of the virus.

In this study, we report for the first time that during the pre‐latency phase of EBV infection, *EBNA2* is m^6^A‐modified, and its expression can be modulated by m^6^A machinery. EBV infection can change the m^6^A abundances on multiple cellular genes such as *FAS* and *TLR9*, which are involved in apoptosis and immune response. We speculate that EBV and host may use m^6^A machinery to interfere with the virus–host interaction, and achieve long‐term latency (such as modulating *TLR9, FAS*, and *EBNA2* m^6^A levels). This study now provides a deeper understanding of EBV–B‐cell interaction at the m^6^A modification level and suggests a critical role for m^6^A modification in EBV infection.

## AUTHOR CONTRIBUTIONS

Xiang Zheng, Jia Wang, Qun Yan, and Jian Ma designed and conceived the experiments. Xiang Zheng, Jia Wang, Xiaoyue Zhang, Qiu Peng, Lingyu Wei, Zhengshuo Li, Can Liu, and Yangge Wu performed the experiments and analyzed the data. Jianhong Lu, Qun Yan, and Jian Ma analyzed data. Xiang Zheng, Jia Wang, Qun Yan, and Jian Ma wrote the paper.

## CONFLICT OF INTERESTS

The authors declare that there are no conflict of interests.

## ETHICS STATEMENT

Collections and use of two health donors' blood samples were approved by the ethical review committees of Central South University and were in accordance with the Declaration of Helsinki. Written informed consent was obtained from the two donors.

## Supporting information

Supporting information.

Supporting information.

## Data Availability

The raw sequencing data obtained from the MeRIP‐seq reported in this study have been deposited in NCBI GEO under accession No. GSE133936.
