# Supplementary material for: RNA m6A methylation regulates virus–host interaction and EBNA2 expression during Epstein–Barr virus infection
Source: Immun Inflamm Dis. 2021 Jan 12;9(2):351–62. doi: 10.1002/iid3.396 (PMC8127537; doi:10.1002/iid3.396)
Supplement: Supplementary file 1 — Supporting information. [file IID3-9-351-s002.pdf]

**Supplementary Figures**

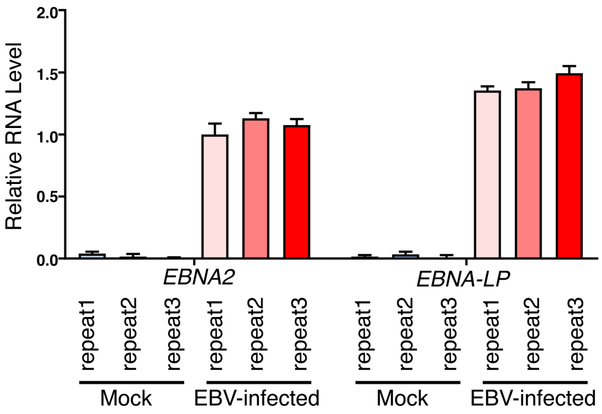


**Supplementary Figure S1.** EBNA2 and EBNA-LP expression in EBV-infected BJAB cells. BJAB cells were infected by EBV (or mock infection as negative control) for 24 h. RT-qPCR analysis of RNAs harvested from cells. *ACTIN* was used as internal control.

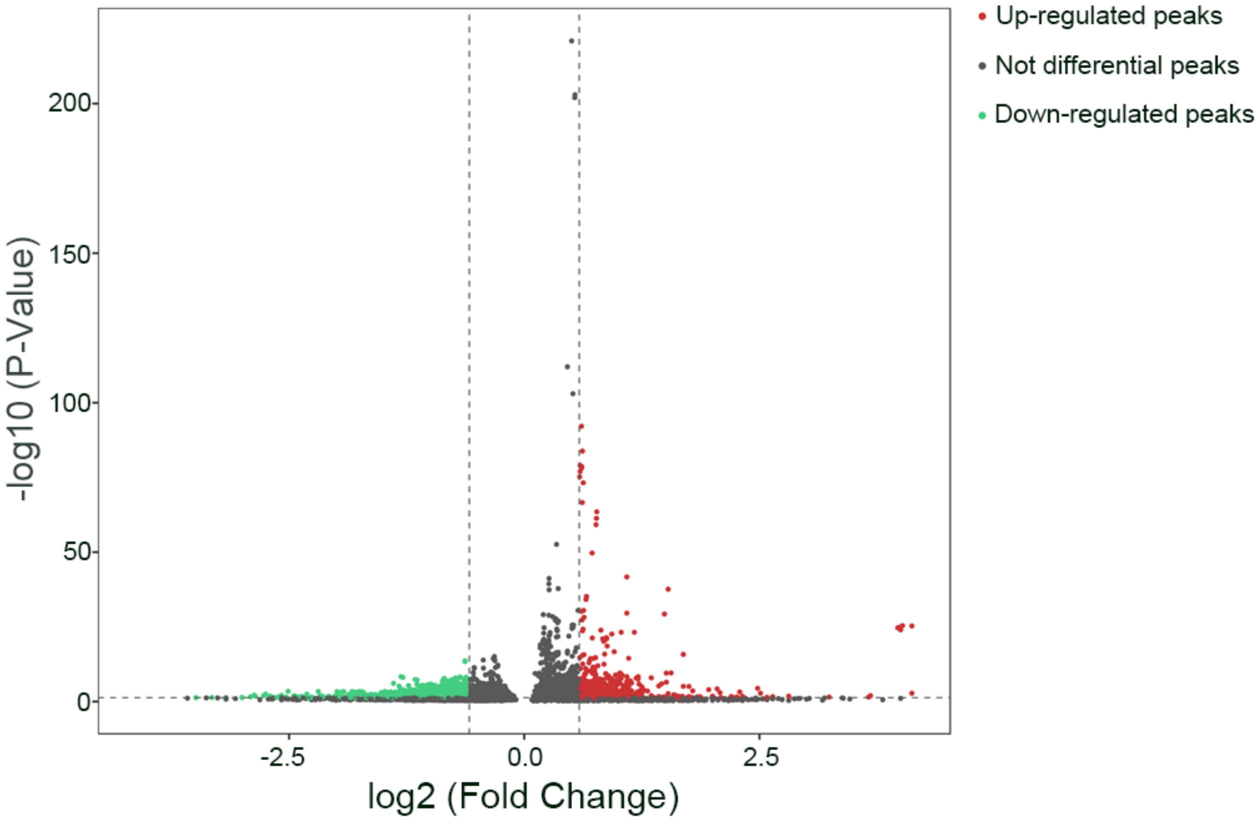


**Supplementary Figure S2.** Volcanic map of m6A modification peaks (left is downregulated, right is upregulated by EBV infection). There are 2586 significantly downregulated m6A modification peaks (from 1046 genes), and 918 significantly upregulated m6A modification peaks (from 416 genes) induced by EBV infection (i.e. EBV infection vs mock). Representative peaks of *TLR9* and *FAS* are shown. The m6A peaks information is included in our MeRIP-seq data (NCBI #GSE133936).

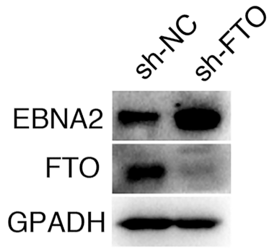


**Supplementary Figure S3.** Raji cells were infected by sh-RNA virus to target FTO expression. EBNA2 and FTO protein levels of Raji cells (sh-NC and sh-FTO) were assayed by Western blotting.

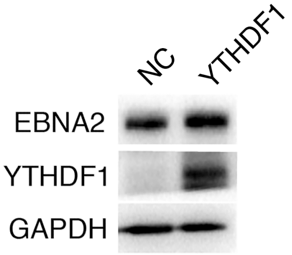


**Supplementary Figure S4.** HEK-293 cells were transfected by either (1) EBNA2 and an empty vector (i.e.NC) or (2) EBNA2 and YTHDF1 expression vector for 48 h. The indicated proteins levels were assayed by Western blotting.

**Supplementary Table S1.** shRNA sequence (5' to 3' )

| shRNA | sequence (5' to 3' ) |
|---|---|
| sh-NC | TTCTCCGAACGTGTCACGT |
| sh-METTL3 | GCTCAACATACCCGTACTACA |
| sh-YTHDF1 | GGATACAGTTCATGACAAT |
| sh-YTHDF2 | CTGCTTATCGTTCCATGAA |
| sh-YTHDF3 | CAATTCAAGGGACACTCAA |

**Supplementary Table S2.** Primers for qPCR

| Primer | Sequence (5' to 3') |
|---|---|
| *IKBKB-F* | CTGGCCTTTGAGTGCATCAC |
| *IKBKB-R* | CGCTAACAACAATGTCCACCT |
| *TLR9-F* | CTGCCACATGACCATCGAG |
| *TLR9-R* | GGACAGGGATATGAGGGATTTGG |
| *UBR4-F* | TCCTACTCCGCCTTCGAGATG |
| *UBR4-R* | CTGAAGTTGGTTCCGGGGAAT |
| *FAS-F* | TCTGGTTCTTACGTCTGTTGC |
| *FAS-R* | CTGTGCAGTCCCTAGCTTTCC |
| *PSMD6-F* | CCTTGTGCAAATCCCTCGACT |
| *PSMD6-R* | AGCTCCTCATCCAAACGCTTC |
| *EBNA2-F* | TCTGCCACCTGCAACACTAA |
| *EBNA2-R* | GTCTGGCACATGCAAGACA |
| *LMP1-F* | AGGCTAGGAAGAAGGCCAAA |
| *LMP1-R* | CTGTTCATCTTCGGGTGCTT |
| *EBNA-LP-F* | TCCCCTCGGACAGCTCCTA |
| *EBNA-LP-R* | CCGCTTACCACCTCCTCTTCT |
| *ACTIN-F* | GAGCTACGAGCTGCCTGACG |
| *ACTIN-R* | GTAGTTTCGTGGATGCCACAG |
| *EBNA2-Pri 1-F* | TGGGGGACAAACATATCATCTA |
| *EBNA2-Pri 1-R* | GTGGAATTAATGGAGTGTCTGA |
| *EBNA2-Pri 2-F* | ATGCCTGGACACAAGAGCCA |
| *EBNA2-Pri 2-R* | CCATCCAAAGCATTCGCATAGC |
| *EBNA2-Pri 3-F* | ACCCTCTAAGACTCAAGGCCA |
| *EBNA2-Pri 3-R* | ATGCTAGGACTGGAGGTGTTTG |
| *EBNA2-Pri 4-F* | AGTCCAGTCCTCGGTCTTCAT |
| *EBNA2-Pri 4-R* | GTTGCCGTGTGTGAATTTCTACA |
| *EBNA2-Pri 5-F* | TGGTATCCTCCATCTATAGACCCC |
| *EBNA2-Pri 5-R* | TTGGGATACATTGGTTGCTGGA |
| *BHRF1-Pri 1-F* | ACAAGGGAGATACTGTTAGCCCT |
| *BHRF1-Pri 1-R* | AGAACTACAGTGTCCTCTGGC |
| *BHRF1-Pri 2-F* | CTGCAGGACATTGTGTTGTAA |
| *BHRF1-Pri 2-R* | CTAACATCCCACGAACTGACA |
| *BHRF1-Pri 3-F* | GGTTGGATTCATCAACAGGGC |
| *BHRF1-Pri 3-R* | GTCAGTCCAGCAAGAAACAAAG |
